# Effects of STAMP Techniques and Conventional Carving on Marginal Adaptation of Class I Composite Resin Restorations: An In Vitro Study

**DOI:** 10.3390/dj14090554

**Published:** 2026-09-02

**Authors:** Benjamin Allen Bingham, Suchunya Rose Bumrung, Hassan Ziada, Neamat Hassan Abubakr

**Affiliations:** 1 Clinical Science Department, School of Dental Medicine, University of Nevada, 1001 Shadow Lane, Las Vegas, NV 89106, USA; binghb2@unlv.nevada.edu (B.A.B.); bumrung@unlv.nevada.edu (S.R.B.); 2 Biomedical Sciences Department, School of Dental Medicine, University of Nevada, Las Vegas, NV 89106, USA; neamat.hassan@unlv.edu

**Keywords:** class I restoration, composite resin, marginal gap, occlusal matrix, STAMP technique, thermal aging

## Abstract

**Background**: Accurate reproduction of occlusal anatomy is a critical determinant of the clinical performance of posterior composite restorations. The present study compares marginal adaptation in Class I composite restorations completed using a Kool-Dam STAMP, a flowable-composite STAMP, or conventional hand-carving techniques. **Methods**: Sixty extracted caries-free molars were randomly allocated to three experimental groups (n = 20). Two fixed marginal sites per tooth (mesial and distal) were measured at four measurement time points: immediately after restoration, before thermocycling, and after 10,000, 30,000, and 50,000 cumulative thermal cycles (5 °C/55 °C; 20 s dwell time). The two site-level values were averaged within each tooth for the primary analysis. A linear mixed-effects model included technique, measurement time point, and the technique-by-time-point interaction, with a random intercept for tooth. **Results**: No significant interaction was observed between restorative technique and measurement time point, indicating that changes in marginal gap over time were not statistically significant among the three techniques (likelihood-ratio χ^2^(6) = 1.43, *p* = 0.964). The overall effect of the technique was also not significant (χ^2^(2) = 1.26, *p* = 0.532). In contrast, marginal-gap measurements increased considerably across the four measurement time points (χ^2^(3) = 218.52, *p* < 0.001), indicating progressive increases in marginal gaps with thermal ageing. Following the Holm adjustment, no pairwise comparisons between techniques at any individual time point achieved statistical significance. **Conclusions**: In the present in vitro study, no statistically significant differences in marginal-gap values were detected among the Kool-Dam STAMP, flowable-composite STAMP, and conventional hand-carving techniques. The study was not designed to demonstrate equivalence or noninferiority; therefore, the absence of a statistically significant difference should not be interpreted as evidence that the techniques are equivalent. These findings suggest that the STAMP approach may be a useful technique for reproducing occlusal morphology while maintaining marginal fit. However, further clinical studies are needed to confirm its long-term performance in vivo.

## 1. Introduction

Reconstruction of occlusal morphology is a critical step in direct posterior composite restoration. Freehand conventional carving remains a widely used method for restoring occlusal form; however, the final morphology is highly technique-sensitive and may require additional adjustments and finishing. Preoperative occlusal matrices have been described as a means of reproducing established anatomy and reducing the need for extensive post-placement carving [1,2,3].

One occlusal index technique was introduced to assist restoration of occlusal cavity preparations by recording intact preoperative occlusal anatomy [1,2]. A clear polyvinyl siloxane (PVS) index is fabricated from the intact occlusal surface before cavity preparation. After placement of the restoration material, the polyvinyl siloxane (PVS) index is placed over the final composite increment to guide the reproduction of the original morphology [2].

Another technique, the occlusal STAMP, uses a preoperative resin-based composite (RBC) to record the intact occlusal anatomy. This fabricated STAMP is then repositioned over the final occlusal composite increment to reproduce the preoperative surface [4,5]. Technical reports and case reports described the technique for selected teeth in which the preoperative occlusal anatomy is sufficiently intact to provide a reliable index [4,5]. Appropriate case selection is therefore essential for the effective application of this technique. It is primarily indicated for Class I lesions with intact occlusal and cusp morphology, where preoperative anatomy provides a clinically useful template [3,4,5]. Although proposed advantages include enhanced anatomical reproduction and reduced contouring requirements, these procedural benefits alone do not establish superior marginal adaptation.

In an exploratory in vitro comparison, Zotti et al. reported no statistically significant differences in microleakage, marginal adaptation, or filling defects between STAMP and conventional restorations. The STAMP group showed larger overflowing margins, longer total restoration time, and shorter finishing time [6]. Furthermore, the material and separating medium used for STAMP fabrication can influence the reproduction of occlusal morphology [7]. Although accurate reproduction of occlusal anatomy is clinically desirable, the relationship between the method used to shape the occlusal surface and the marginal adaptation of the restoration remains uncertain. A preoperative occlusal index or STAMP may facilitate anatomical reproduction and reduce the need for extensive contouring or occlusal adjustment. However, pressure applied during STAMP seating, displacement of the uncured composite, the material used to fabricate the STAMP, and subsequent finishing procedures may influence the restoration margin. Therefore, procedural convenience or improved anatomical reproduction should not be assumed to produce superior marginal adaptation.

The available literature on the STAMP technique consists predominantly of technical descriptions, case reports, and a limited number of laboratory comparisons. Previous studies have evaluated outcomes such as occlusal reproduction, microleakage, restoration defects, operative time, and marginal quality; however, evidence concerning the progression of marginal gaps during cumulative thermal aging remains limited. Furthermore, few investigations have directly compared different STAMP materials with conventional hand-carving under standardized restorative and aging conditions. This represents an important gap because thermal changes may progressively affect the tooth–restoration interface, regardless of the technique used to reproduce the occlusal surface.

Direct comparison of restorative techniques also presents several methodological challenges. Cavity dimensions, restorative and adhesive materials, composite placement, curing procedures, operator-related variability, STAMP seating pressure, finishing and polishing, measurement location, and aging conditions must be controlled to isolate the effect of the occlusal-shaping technique. Marginal-gap measurements obtained from multiple sites and at repeated time points within the same tooth are also correlated and should not be treated as independent observations. The present study therefore used standardized cavity preparations and restorative procedures, fixed measurement sites, cumulative thermal aging, and a repeated-measures statistical model that accounted for clustering within teeth.

This study addresses two research questions: (1) whether marginal-gap measurements differed among conventional hand-carving, Kool-Dam STAMP, and flowable-composite STAMP techniques; and (2) whether the marginal-gap trajectory across the four measurement time points differed among techniques. The null hypothesis was that marginal-gap measurements over the four measurement time points would not differ among the three techniques.

The aim of this study was to evaluate and compare marginal-gap measurements of Class I (RBC) restorations fabricated with two occlusal STAMP materials and conventional hand-carving at four measurement time points during laboratory thermal ageing.

## 2. Materials and Methods

### 2.1. Study Design and Specimens

Sixty extracted, caries-free posterior teeth (maxillary and mandibular molars) with intact occlusal morphology were selected for this in vitro study. The teeth were obtained from patients treated at the Oral Surgery Department of the University of Nevada, Las Vegas School of Dental Medicine. The use of extracted teeth for research purposes was covered by the standard consent procedure for patients undergoing extraction. The ethical principles for the collection and use of these teeth were observed in accordance with the Declaration of Helsinki. The Institutional Review Board of the University of Nevada, Las Vegas determined that the use of de-identified extracted teeth did not constitute human-subjects research (IRB approval #: UNLV-2026-397).

The inclusion criteria were teeth with intact occlusal surfaces, no caries or restorations, and no visible cracks or stains. Teeth with fractures, previous restorations, caries, cavities, cracks, or stains were excluded. The selected teeth were disinfected and stored in artificial saliva (Pickering Laboratories, Mountain View, CA, USA) at room temperature. During experimental procedures, specimens were embedded upright in polyvinyl siloxane (VPS; Kerr Extrude XP, Kerr, Romulus, MI, USA) to support standardized positioning.

The sample-size calculation was performed a priori using G*Power version [3.1.9]. The calculation was based on a fixed-effects one-way omnibus analysis of variance (ANOVA) with three experimental groups. An effect size of Cohen’s f = 0.54, derived from Klein et al. [7], was used, together with a significance level of α = 0.05 and a target statistical power of 1 − β = 0.95. Under these assumptions, the minimum required total sample size was 57 teeth. The sample size was increased to 60 teeth to permit equal allocation of 20 teeth per group and to allow for possible specimen loss or damage. Eligible teeth were randomly allocated to three groups (n = 20 teeth per group):

Group 1, Kool-Dam Heatless Liquid Dam STAMP (Pulpdent, Watertown, MA, USA);
Group 2, Flowable-composite STAMP (3M, St. Paul, MN, USA); and
Group 3, Conventional hand-carving.

For the allocation sequence, the 60 eligible teeth were assigned in a 1:1:1 ratio to the Kool-Dam STAMP, flowable-composite STAMP, or conventional hand-carving group. Allocation was implemented using a sealed opaque envelope.

### 2.2. STAMP Fabrication and Cavity Preparation

**Figure 1:**
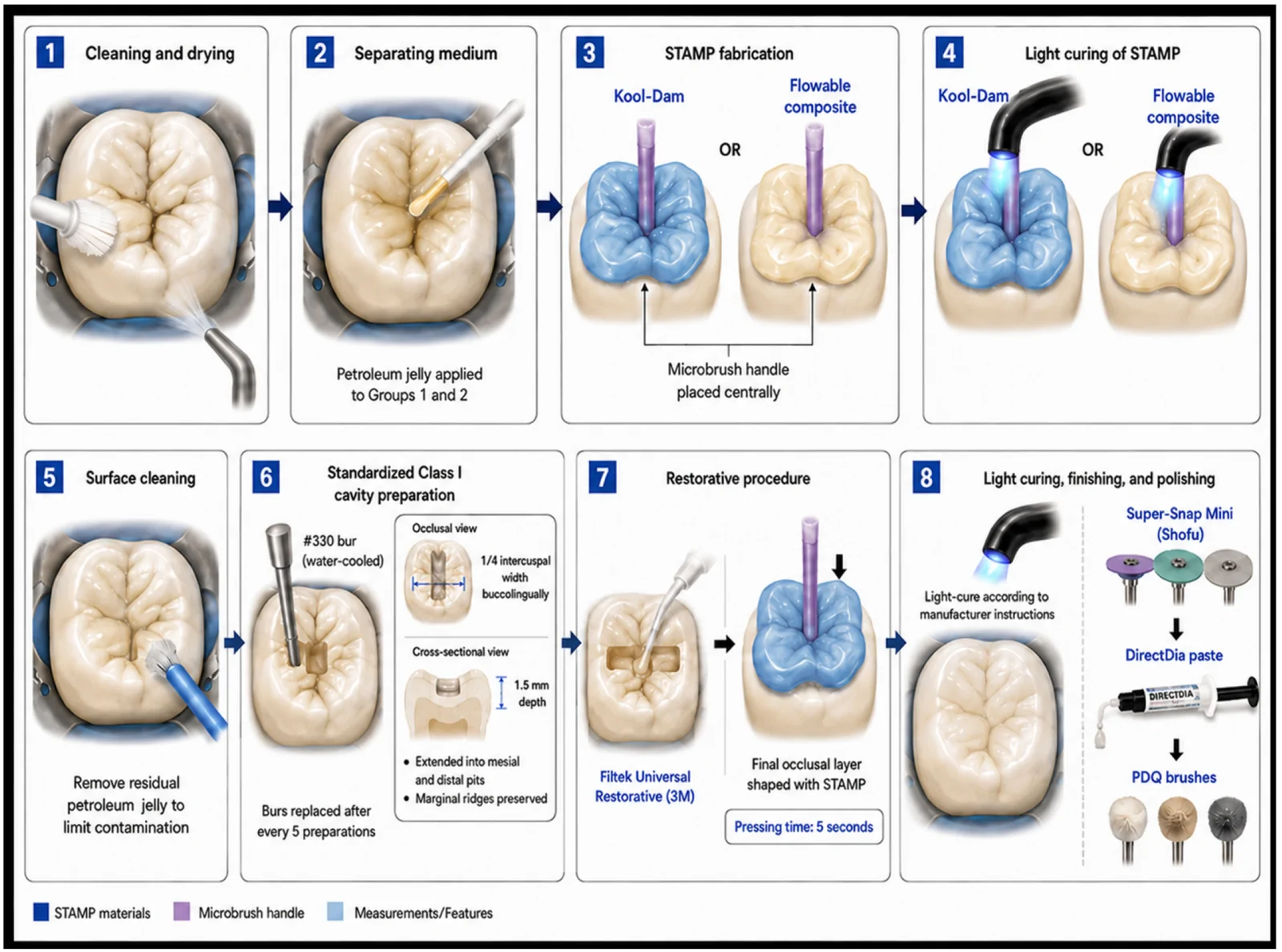
Schematic representation of STAMP fabrication and restoration workflow (AI-generated).

Teeth were cleaned and dried. In the two STAMP groups, the intact occlusal surface was initially coated with a thin layer of petroleum jelly during the STAMP fabrication. A microbrush was incorporated as a handle for placement and removal of the STAMP. The STAMP material was light-cured according to the relevant manufacturer’s instructions and retained for use during restoration. Tooth surfaces were then cleaned to remove residual separating medium before bonding (Figure 1).

Standardized Class I cavity preparations were prepared with a water-cooled #330 bur (Komet USA, Rock Hill, SC, USA) to a depth of 1.5 mm and approximately one-quarter of the intercuspal buccolingual width. Preparations extended into the mesial and distal pits while preserving the marginal ridges. Burs were replaced after every five preparations. All cavity preparations and restorations were completed by two operators who also performed the profilometric measurements. These measurements were performed by the same operators, blinded to the restorative technique, using coded specimen identifiers. The preparations were treated with 3M ESPE Scotchbond Universal Adhesive (3M ESPE, Oral Care Solutions Division, St. Paul, MN, USA) according to the manufacturer’s instructions. 3M ESPE Scotchbond Universal Etchant (3M ESPE, Oral Care Solutions Division, St. Paul, MN, USA) was applied for 15 s, followed by a 15 s rinse and controlled drying. The adhesive was applied to the preparation and scrubbed in for 20 s. The adhesive was lightly air-dried for 5 s to facilitate solvent evaporation before being light-cured for 10 s using the VALO LED curing light (Ultradent Products, South Jordan, UT, USA). Filtek Universal Restorative composite was inserted using incremental placement with a maximum increment thickness of 2 mm. All restorations were fabricated using Filtek Universal Restorative composite (3M) for standardization purposes.

For Groups 1 and 2, the final occlusal composite layer was shaped with the fabricated STAMP using a 5 s seating time. All restorations were light-cured according to the manufacturer’s instructions, finished with Super-Snap Mini (Shofu), and polished with DirectDia paste and PDQ brushes. Light curing was performed using the VALO LED curing light (Ultradent Products, South Jordan, UT, USA) at an irradiance of 1000 mW/cm^2^ for 10 s per 2 mm increment. Irradiance was verified using Radiometer X (SDI, North America, Itasca, IL, USA) in the frequency range of 550 nm.

### 2.3. Marginal-Gap Measurement and Thermal Aging

The marginal adaptation assessment was blinded for the technique used and measured at two fixed points per tooth, coded M and D, using a 3D non-contact profilometer (VR-3100; Keyence, Osaka, Japan). Before measurement, the profilometer was calibrated according to the manufacturer’s instructions. Each specimen was positioned using the standardized positioning method. The mesial and distal sites were identified using established anatomical landmarks and evaluated at 40× magnification. At each site, the marginal gap was defined as the vertical distance between the tooth structure and the restoration interface at a constant, predetermined location for each tooth. A single measurement was obtained at each mesial and distal site and recorded for subsequent statistical analysis. Data were analyzed using IBM SPSS Statistics (version 23; IBM Corp., Armonk, NY, USA). Measurements were recorded and analyzed in micrometres (µm). Lower values indicated better marginal adaptation.

Thermocycling was performed in a thermocycler (SD Mechatronik, Feldkirchen-Westerham, Germany) between 5 °C and 55 °C with 20 s dwell times to simulate thermal aging [8].

Marginal-gap measurements were obtained at four time points: immediately after restoration before thermocycling, and after 10,000 (1 estimated clinical year), 30,000 (3 estimated clinical years), and 50,000 cumulative thermal cycles (5 estimated clinical years). These intervals were treated as laboratory aging points and were not interpreted as validated equivalents of clinical service time.

### 2.4. Statistical Analysis

The experimental unit was the tooth. Because each tooth contributed two site-level measurements at each measurement time point, mesial and distal readings were not treated as independent specimens. For the primary analysis, the two site-level values were averaged within each tooth at each measurement time point, yielding one tooth-level marginal-gap score per time point. All 60 teeth had complete measurements at all four time points.

A linear mixed-effects model was fitted using maximum likelihood, with restorative technique, measurement time point, and the technique-by-measurement-time-point interaction as fixed effects and a random intercept for tooth. Nested likelihood-ratio tests evaluated the interaction, technique main effect, and measurement-time-point main effect. Model-estimated between-technique contrasts at each time point were reported with 95% confidence intervals, with *p* values adjusted across the 12 contrasts using Holm’s procedure.

As a sensitivity analysis, the original mesial and distal site-level measurements were analyzed using generalized estimating equations (GEEs), with tooth specified as the clustering unit to account for within-tooth correlation. A Gaussian distribution with an identity link, an exchangeable working correlation structure, and robust standard errors was used. Restorative technique, measurement time point, and their interaction were evaluated using Wald-chi-square tests.

## 3. Results

The study included 120 fixed measurement sites: two sites, mesial and distal, on each of 60 teeth. Each site was evaluated at four measurement time points, resulting in 480 site-level observations. No outcome values were missing, and no observations were excluded from the analysis.

### 3.1. Descriptive Outcomes

Table 1 presents tooth-level mean marginal-gap values, calculated as the average of the two fixed-site measurements for each tooth. Across all three techniques, mean marginal-gap values increased from immediately after restoration to after 50,000 cumulative thermal cycles. Hand-carving showed descriptively higher mean values at each measurement time point; however, the mixed-effects model did not detect a statistically significant overall technique effect or a statistically significant difference in trajectories among techniques.

### 3.2. Primary Repeated-Measures Analysis

The technique-by-measurement-time-point interaction was not statistically significant (likelihood-ratio χ^2^(6) = 1.43, *p* = 0.964), indicating no evidence that marginal-gap trajectories differed among the three restorative techniques. In the additive model, there was no overall technique effect (χ^2^(2) = 1.26, *p* = 0.532), whereas the measurement-time-point effect was statistically significant (χ^2^(3) = 218.52, *p* < 0.001; Table 2). Relative to immediately after restoration, before thermocycling, the model-estimated average increases across techniques were 2.2 µm (95% CI, 1.5 to 3.0) after 10,000 cycles, 5.0 µm (95% CI, 4.3 to 5.8) after 30,000 cycles, and 7.5 µm (95% CI, 6.7 to 8.2) after 50,000 cycles.

**Figure 2:**
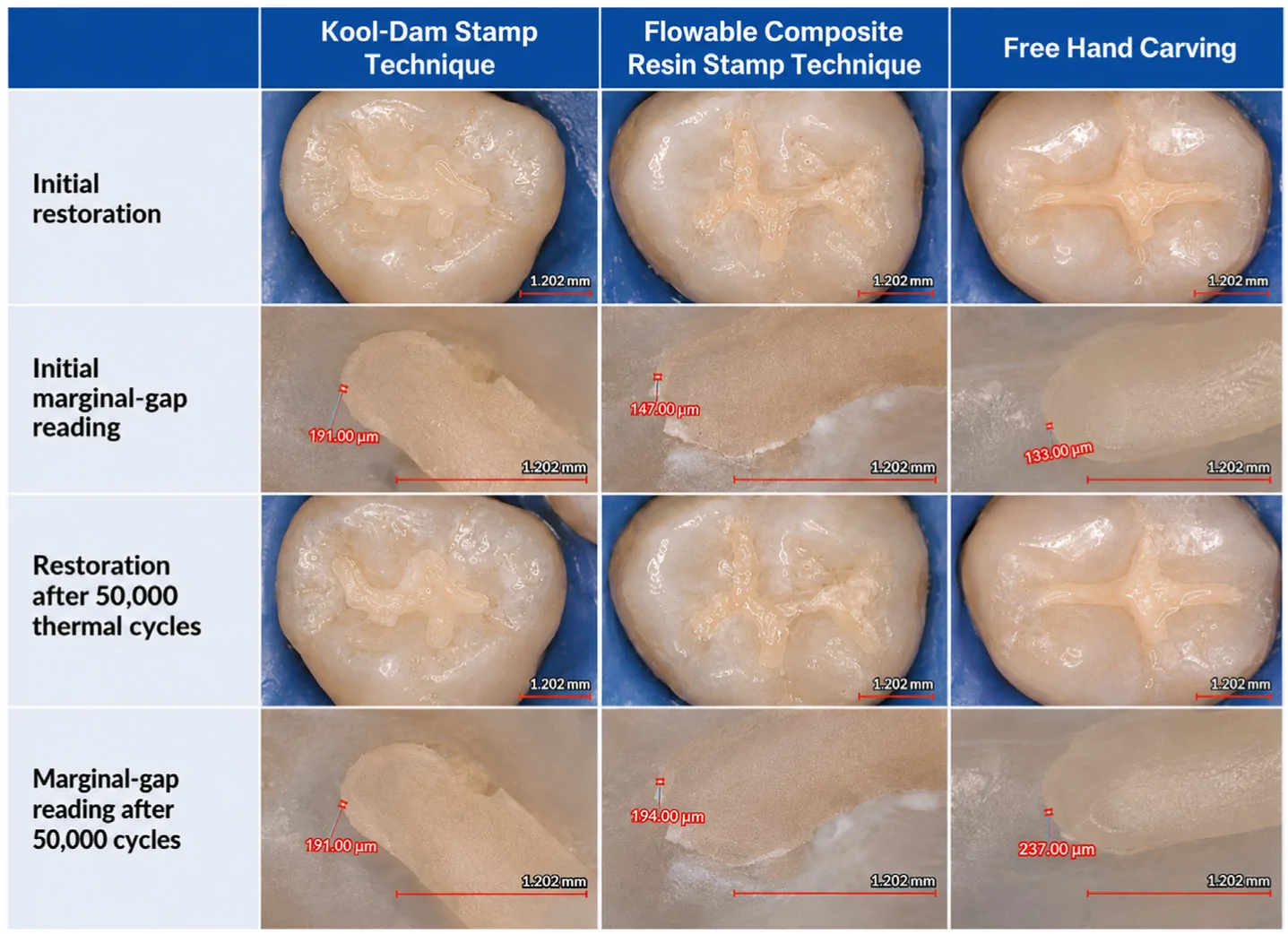
Representative occlusal and marginal-gap images for the three restorative techniques.

Representative images are shown for the initial restoration and after 50,000 cumulative thermal cycles (Figure 2).

No model-estimated pairwise technique contrast at any measurement time point was statistically significant after Holm adjustment (all adjusted *p* values = 1.000; Table 3).

The site-level generalized estimating equation (GEE) sensitivity analysis yielded findings consistent with the primary tooth-level analysis. The restorative-technique-by-measurement-time-point interaction was not statistically significant (Wald χ^2^(6) = 2.60, *p* = 0.858), and no statistically significant overall effect of restorative technique was detected (Wald χ^2^(2) = 0.94, *p* = 0.626). In contrast, the effect of measurement time point was statistically significant (Wald χ^2^(3) = 220.73, *p* < 0.001), indicating that the principal conclusions were unchanged when the mesial and distal site-level observations were analyzed separately while accounting for within-tooth correlation.

**Figure 3:**
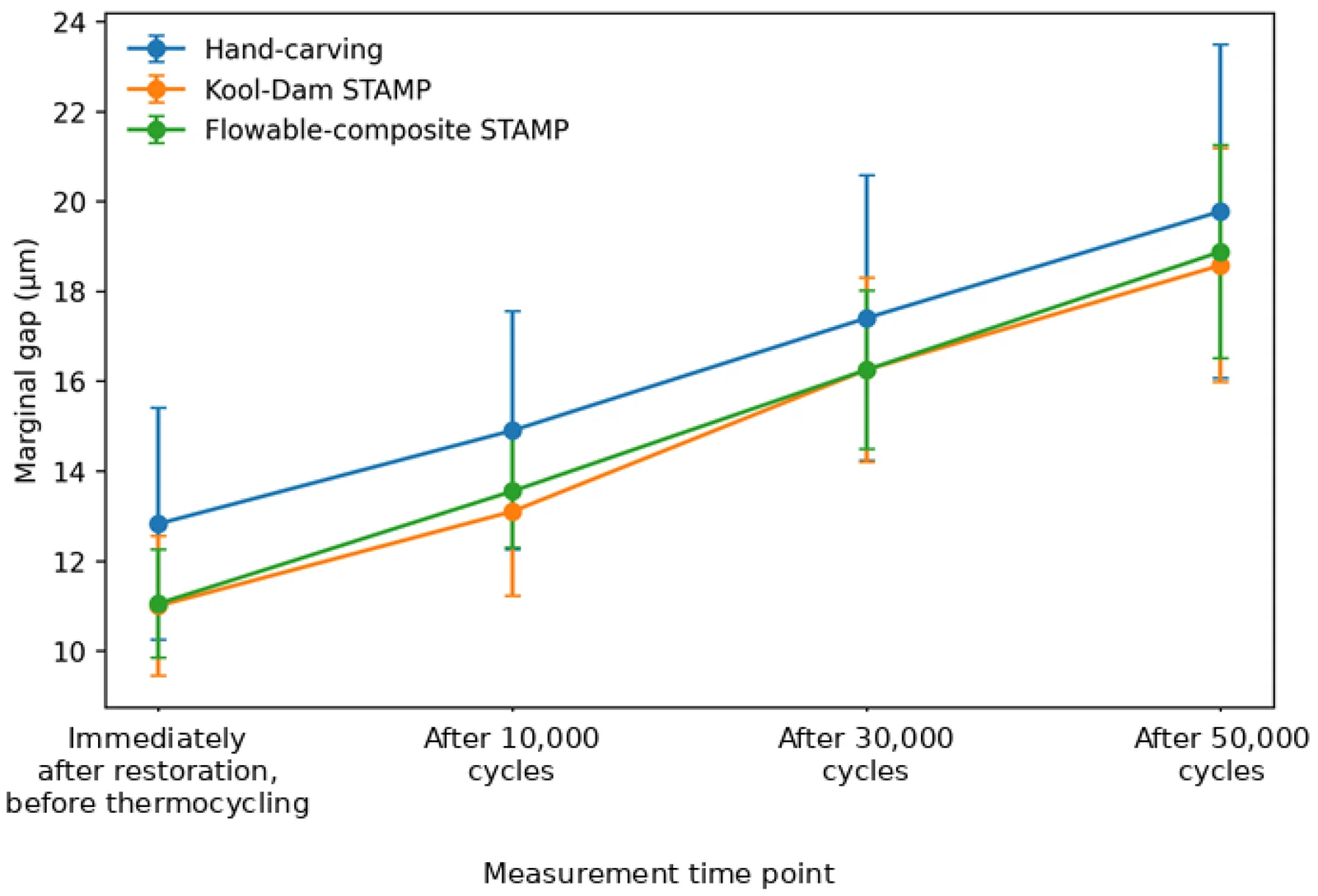
Tooth-level mean marginal-gap values by restorative technique and measurement time point. Error bars show 95% confidence intervals around group means (n = 20 teeth per group). Values are reported in µm.

Figure 3 illustrates the increase in mean marginal-gap values across measurement time points for all three restorative techniques, with broadly similar trajectories among groups.

## 4. Discussion

After adjustment for clustering and repeated observations, the study did not demonstrate that either occlusal STAMP material produced lower marginal-gap values than conventional hand-carving. The absence of a technique-by-measurement-time-point interaction indicates that the increase observed across cumulative thermal cycles was not detectably different among techniques. These findings should be interpreted as a failure to detect a difference under the present laboratory conditions, rather than as evidence of equivalence between the approaches.

The present findings are consistent with the exploratory study by Zotti et al., who reported no statistically significant differences in marginal adaptation, microleakage, or restoration defects between STAMP and conventionally shaped restorations [6]. Their study also found that the STAMP technique was associated with larger overflowing margins, a longer total restoration time, and a shorter finishing time. Although these observations suggest that the STAMP technique may alter procedural workflow, the present study did not assess restoration time, excess material, finishing duration, internal defects, or microleakage. Direct comparison of these secondary outcomes is therefore not possible.

Bud et al. compared techniques used to create occlusal anatomy and evaluated microleakage in direct occlusal restorations [9]. Their findings contribute to the evidence that the method used to shape the occlusal surface may influence some aspects of restoration performance. However, microleakage and surface marginal-gap measurements are not identical outcomes. Microleakage evaluates penetration along the tooth–restoration interface, whereas the non-contact profilometry used in the present study quantified the external marginal gap at predetermined sites. Differences in outcome definition, measurement method, cavity preparation, restorative protocol, and ageing conditions may therefore explain variation among studies.

Klein et al. demonstrated that the material and separating medium used for STAMP fabrication can influence the accuracy with which occlusal anatomy is reproduced [7]. Their findings highlight the technique sensitivity of the STAMP fabrication and support the need to standardize the material and clinical workflow. Nevertheless, accurate reproduction of occlusal anatomy does not necessarily imply improved marginal adaptation. The present study specifically evaluated marginal-gap values during cumulative thermal ageing and detected no statistically significant differences between the Kool-Dam STAMP, flowable-composite STAMP, and conventional hand-carving techniques.

Taken together, the available evidence suggests that STAMP techniques may provide practical advantages in reproducing preoperative occlusal morphology, but their effects may depend on the outcome being evaluated. Differences among published studies may also reflect variation in STAMP materials, separating media, composite placement, seating pressure, finishing procedures, measurement techniques, thermal-cycling protocols, and the inclusion or exclusion of mechanical loading. Standardized studies incorporating both marginal and anatomical outcomes are therefore needed before firm conclusions regarding the relative advantages of individual STAMP techniques can be drawn.

The present study did not measure operative time, occlusal adjustment, overhangs, internal voids, microleakage, or reproduction of occlusal anatomy; therefore, it cannot confirm or refute those procedural effects.

Marginal outcomes in RBC restorations are affected by factors beyond the method used to reproduce final occlusal anatomy [9]. Aqueous ageing can contribute to hydrolytic degradation at the resin-dentine interface [10]. Thermal expansion characteristics can also contribute to stresses in the tooth-restoration complex during temperature change [11], and the influence of thermal cycling on marginal integrity varies with restorative material and cycling conditions [12]. Technique-sensitive variables, including handling, polymerization, and dimensional change, remain important considerations [13].

The model detected higher marginal-gap values across the four measurement time points. The cumulative thermal-cycle counts represent laboratory ageing conditions rather than clinical follow-up time. The study also did not include mechanical loading. Cyclic occlusal loading has been associated with greater microleakage in a Class I RBC-restoration model and should be considered in future experiments [14].

From a clinical perspective, restoration longevity is multifactorial and cannot be inferred from a single laboratory marginal-gap outcome [15,16]. The STAMP technique may be selected for teeth with suitable intact morphology or to facilitate workflow; however, the present study does not demonstrate a marginal-adaptation advantage, equivalence, noninferiority, or clinical performance of either STAMP material relative to conventional hand-carving.

The dataset included 20 teeth per group and was not designed as an equivalence or noninferiority study with a clinically justified margin. Therefore, non-significant between-group tests do not establish equivalence.

### Limitations

Several limitations should be noted in this study. First, the research was conducted under in vitro conditions, utilizing thermocycling as the sole thermal ageing method. Consequently, other clinically relevant factors, including mechanical fatigue, pH fluctuations, biofilm formation, pulpal pressure, and functional occlusal loading, were not simulated. Second, marginal adaptation was evaluated at two predetermined sites per tooth rather than along the entire tooth–restoration interface. While this approach enhanced standardization and reproducibility, it may not fully reflect regional variations across the complete cavosurface margin. Third, maxillary and mandibular molars were analyzed collectively without stratification by dental arch, tooth type, or detailed occlusal morphology, which could have influenced the results. In addition, only one restorative composite and one finishing/polishing protocol were assessed, thereby limiting the generalizability of the findings to other materials, adhesive systems, and clinical procedures. Overall, the two-point non-contact three-dimensional profilometry protocol provided an effective balance between standardized, reproducible measurement and comprehensive marginal assessment.

## 5. Conclusions

Within the limitations of this in vitro study, no statistically significant differences in marginal-gap values were detected among the Kool-Dam STAMP, flowable-composite STAMP, and conventional hand-carving techniques at any of the four measurement time points. Marginal-gap values increased across the four measurement time points in all groups, with no evidence that the pattern of change differed among techniques. Because the study was not designed as an equivalence or noninferiority investigation, these findings should not be interpreted as demonstrating equivalence among the techniques.

## Figures and Tables

**Table 1:** Tooth-level mean (SD) marginal-gap values by restorative technique and measurement time point.

Measurement Time Point	Hand-Carving (n = 20)	Kool-Dam STAMP (n = 20)	Flowable-Composite STAMP (n = 20)
Immediately after restoration, before thermocycling	12.8 (5.5)	11.0 (3.3)	11.1 (2.6)
After 10,000 cumulative thermal cycles	14.9 (5.7)	13.1 (4.0)	13.6 (2.7)
After 30,000 cumulative thermal cycles	17.4 (6.8)	16.2 (4.4)	16.2 (3.8)
After 50,000 cumulative thermal cycles	19.8 (7.9)	18.6 (5.6)	18.9 (5.1)

Values are mean (SD), µm. The two fixed-site measurements were averaged within each tooth (n = 20 teeth per group).

**Table 2:** Primary tooth-level repeated-measures mixed-effects analysis.

Effect	Likelihood-Ratio Test	*p* Value	Interpretation
Technique × measurement time point	χ^2^(6) = 1.43	0.964	No evidence of different trajectories
Technique	χ^2^(2) = 1.26	0.532	No overall technique effect detected
Measurement time point	χ^2^(3) = 218.52	<0.001	Marginal-gap values increased over time

Model: fixed effects for restorative technique, measurement time point, and technique × measurement time point; random intercept for tooth. Likelihood-ratio tests were fitted using maximum likelihood.

**Table 3:** Model-estimated between-technique contrasts at each measurement time point.

Measurement Time Point	Comparison	Difference (95% CI), µm
Immediately after restoration, before thermocycling	Kool-Dam–hand-carving	−1.8 (−4.9 to 1.2)
Immediately after restoration, before thermocycling	flowable-composite–hand-carving	−1.8 (−4.8 to 1.3)
Immediately after restoration, before thermocycling	Kool-Dam–flowable-composite	−0.1 (−3.1 to 3.0)
After 10,000 cumulative thermal cycles	Kool-Dam–hand-carving	−1.8 (−4.8 to 1.2)
After 10,000 cumulative thermal cycles	flowable-composite–hand-carving	−1.3 (−4.4 to 1.7)
After 10,000 cumulative thermal cycles	Kool-Dam–flowable-composite	−0.5 (−3.5 to 2.6)
After 30,000 cumulative thermal cycles	Kool-Dam–hand-carving	−1.1 (−4.2 to 1.9)
After 30,000 cumulative thermal cycles	flowable-composite–hand-carving	−1.1 (−4.2 to 1.9)
After 30,000 cumulative thermal cycles	Kool-Dam–flowable-composite	0.0 (−3.0 to 3.0)
After 50,000 cumulative thermal cycles	Kool-Dam–hand-carving	−1.2 (−4.2 to 1.8)
After 50,000 cumulative thermal cycles	flowable-composite–hand-carving	−0.9 (−3.9 to 2.1)
After 50,000 cumulative thermal cycles	Kool-Dam–flowable-composite	−0.3 (−3.3 to 2.7)

Negative estimates indicate a smaller model-estimated marginal-gap value for the first-named technique. Confidence intervals are unadjusted.

## Data Availability

The original contributions presented in this study are included in the article. Further inquiries can be directed to the corresponding author.
